# Normalization of thyroid function tests among thyrotoxicosis patients attending a University Hospital in North-West Ethiopia

**DOI:** 10.1186/s13044-019-0064-2

**Published:** 2019-03-23

**Authors:** Eyob Alemayehu Gebreyohannes, Emneteab Mesfin Ayele, Soliana Alemayehu Tesfaye, Mohammed Assen Seid

**Affiliations:** Department of Clinical Pharmacy, Lecturer and Preceptor of Clinical Pharmacy, UOG University of Gondar College of Medicine and Health Sciences, School of Pharmacy, Gondar, Ethiopia

**Keywords:** Thyrotoxicosis, Hyperthyroidism, Thyroid, Propylthiouracil, Ethiopia

## Abstract

**Background:**

Thyrotoxicosis is a clinical state that results from inappropriately high thyroid hormone action in tissues. Although it is one of the common endocrine disorders, there is scarcity of data on the management of thyrotoxicosis in Africa, particularly in Ethiopia. The aim of this study was to investigate treatment outcomes and determinants of treatment outcomes among hyperthyroid patients on antithyroid drugs attending a teaching hospital in Ethiopia.

**Methods:**

A retrospective cohort study was conducted on medical records of patients with thyrotoxicosis who had attended the medical inpatient ward and chronic ambulatory clinic of the University of Gondar Comprehensive Specialized Hospital in Ethiopia between June 2013 and April 2018. Descriptive statistics were used to summarize socio-demographic and other baseline information. A cox regression method was used to determine factors associated with normalization of thyroid function tests (TFTs). All statistical tests were performed using STATA version 14.

**Results:**

Data from a total 211 patients were eventually analyzed. The mean age of the patients was 47.25 ± 14.26 years with female majority (94.31%). The most common etiology was toxic multinodular goiter (54.90%). Because methimazole or carbimazole are not easily available locally, all of the patients included in this study were taking propylthiouracil (PTU). Nearly 9 out of 10 patients (90.38%) had symptom resolution within a mean period of 5.37 (± 6.59) months. Sixty-two (29.81%) and 122 (58.65%) patients achieved normalization of TSH and FT4, respectively during the study period. The mean time to normalization of TSH and FT4 was 13 (±13.28) and 11.53 (±13.39) months, respectively. On the other hand, T3 and all three TFTs were normalized only among 79 (38.16%) and 55 (26.32%) patients, respectively. Older age and higher baseline FT4 levels were shown to modestly reduce the chances of achieving normalization of TSH.

**Conclusion:**

Though PTU is not the preferred antithyroid agent in the management of thyrotoxicosis, all patients used PTU for the management of hyperthyroidism. All TFTs were normalized in only less than one-third of the participants. Resolution of symptoms took longer period of time than expected.

## Background

Thyrotoxicosis is a clinical state that results from inappropriately high thyroid hormone action in tissues [1]. It affects 1–3% of the general population and is 10 times more common in women than in men [2]. In the United States, the prevalence of thyrotoxicosis is approximately 1.2% [1]. The extent of thyroid disorders in Africa remains unknown because of under-diagnosis and underreporting but the few available studies note prevalence rate of 1.2 to 9.9% [3]. In Ethiopia, the prevalence of thyroid disease is reported to be 1.2% [3].

Graves’ disease (GD) is the most common cause of thyrotoxicosis. It accounts for 70–80% of cases in iodine-sufficient population and approximately 50% of cases in iodine-deficient areas [4]. Other etiologies include thyroiditis, toxic nodular goiter, toxic multinodular goiter (TMNG), toxic adenoma, and exogenous thyrotoxicosis (iatrogenic, factitious, iodine induced) [5].

Untreated thyrotoxicosis can lead to serious complications such as thyroid storm, arrhythmia, hypertension, cardiac dilation, congestive heart failure (CHF), and sudden cardiac arrest [6, 7]. The three principal treatment options for the management of thyrotoxicosis include thionamides or anti-thyroid drugs (ATDs), radioactive iodine, and surgery [1].

Ethiopia is among iodine-deficient countries in the globe. Even though, the mandatory salt iodization that was implemented in 2011 has led to significant improvements, the country has a long way to go to eradicate iodine deficiency disorders [8]. Despite the availability of studies on pharmacologic treatment outcomes among patients with thyrotoxicosis, most of the previous studies were conducted in other countries and there is scarcity of data in Ethiopia context. To the best of the authors’ knowledge, this is the first study to evaluate pharmacologic treatment outcomes among hyperthyroid patients from Ethiopia. The aim of this study was to investigate rates and determinants of normalization of thyroid function tests (TFTs) among hyperthyroid patients on ATDs attending University of Gondar Comprehensive Specialized Hospital (UoGCSH).

## Methods

### Study area and period

The study was conducted from April 1 to May 15, 2018 at the UoGCSH which is one of the oldest teaching hospitals in Ethiopia that is located in the northwest Ethiopia, 727 km away from the capital city Addis Ababa. The hospital serves as a referral center for an estimated 7 million people most of which come from rural areas with poor socioeconomic status.

### Study design and population

A retrospective cohort study was conducted on medical records of all patients with the diagnosis of thyrotoxicosis who had attended the medical inpatient ward and chronic ambulatory clinic of UoGCSH between June 2013 and April 2018. The chart numbers were entered into Microsoft Office Excel 2013 and checked for duplication. Then data was collected by using chart review of the consecutive hyperthyroid patients.

Management of thyrotoxicosis at UoGCSH is done by internists, resident physicians, and general practitioners. There is no separate local or institutional guideline for the management of thyrotoxicosis but a national standard treatment guideline is available.

### Inclusion and exclusion criteria

#### Inclusion criteria

Patients 18 years of age and older with a diagnosis of thyrotoxicosis who have received antithyroid medications for management of thyrotoxicosis were included in the study.

#### Exclusion criteria

The following patients were excluded from the study: patients who visited either the medical inpatient ward or chronic ambulatory clinic only once and do not have further follow-up history; patients with less than 4 weeks follow-up; patients with incomplete medical records of thyroid function tests (TFTs); pregnant patients; and patients who underwent surgery for the management of thyrotoxicosis. There were no patients who received radioiodine therapy.

### Study variables

#### Primary outcome measure

Normalization of TFTs was considered as primary outcome measures.

#### Secondary outcome measures

Secondary outcome measures included, time to symptom resolution, determinants of treatment outcomes, treatment related adverse effects, and major complications.

### Data quality assurance

Before data collection, the data collection instrument was pretested on 20 samples. Then, the data collection instrument was modified based on the results of the pretest. During data collection, the data on the data collection tool and the medical records was rechecked for accuracy after data collection for each sample. Each variable had been coded before data entry to minimize manual data entry. After data was entered into the STATA version 14, it was cross-checked for accuracy of data entry.

### Data compilation and analysis

Descriptive statistics were used to summarize socio-demographic and other baseline information. Categorical variables were expressed as frequencies (percentage) and quantitative variables as mean ± standard deviation. A cox regression method was used to determine factors associated with normalization of TFTs. All statistical tests were performed using STATA version 14.

### Independent variables

Age, initial PTU dose, maintenance PTU dose, duration of symptoms, baseline TSH, baseline FT4, and baseline T3 were handled as continuous variables while sex, etiologies of thyrotoxicosis, and world health organization (WHO) goiter size were handled as categorical variables.

### Definition of terms and operational definition

**Thyrotoxicosis:** TSH level < 0.4 mU/L [1].

**TSH, FT4 and T3 normalization**: when TSH, FT4 and T3 are within the euthyroid range.

**Euthyroid range:** is considered when TSH, FT4, and T3 values are between 0.4–5.0 mU/L, 10.4–19.6 pmol/l and 0.92–2.3 nmol/l, respectively.

**WHO goiter size:** Grade 0: The goiter is not palpable or visible even when the neck is extended; Grade 1: The goiter is detected on palpation and/or visible when the neck is extended; Grade 2: Goiter is visible when neck is in the normal position; Grade 3: Large goiter visible from distance.

Classification of the different types of thyrotoxicosis was mainly made on clinical grounds (i.e. based on clinical examination) and was simply taken from the medical records. A TMNG is simply a late-stage goiter that’s been around for a while and has had a chance to grow and become lumpy or nodules. Toxic nodular goiter involves an enlarged thyroid gland that contains a small rounded mass or masses called nodules, which produce too much thyroid hormone.

## Results

### Socio-demographic and clinical characteristics

**Fig. 1:**
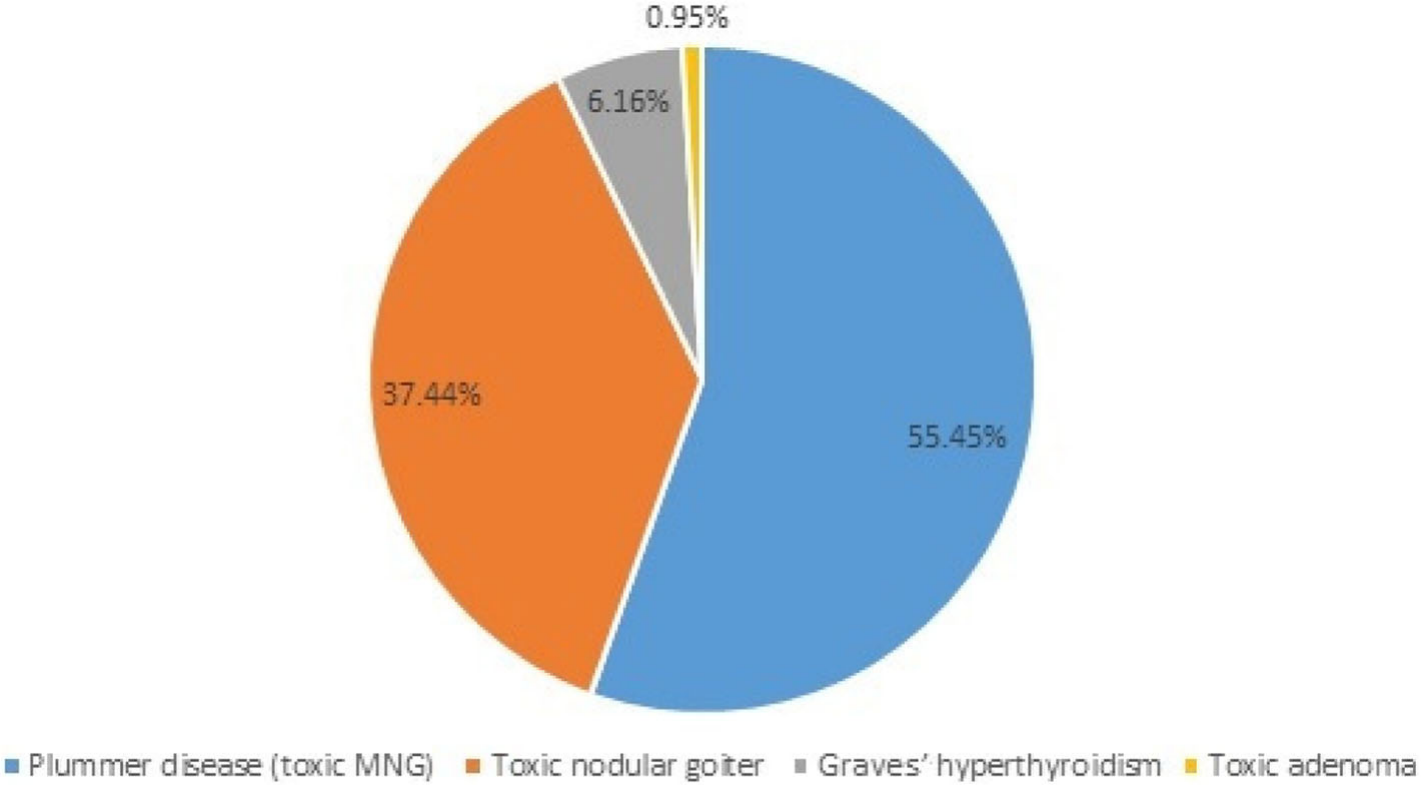
The etiology of thyrotoxicosis among hyperthyroid patients attending UoGCSH (*N* = 211)

Initially, 224 patient medical records were assessed for eligibility of which 13 patients were excluded because they had less than 4 weeks of follow-up (*N* = 4), had incomplete medical records (*N* = 5), were pregnant patients (*N* = 2), and underwent surgery for the management of thyrotoxicosis (N = 2). A total 211 patients were included in the final analysis. The mean age of the patients was 47.25 years. Majority of the patients were female 199 (94.31%). According to WHO goiter size classification, most of the patients (56.22%) were with grade II goiter size. Majority of the patients had baseline FT4 and T3 values of > 100 pmol/l and > 2.3 nmol/l, respectively. The mean (±SD) duration of symptom before seeking treatment was 88.78 (±139.08) months (Table 1). This longer duration was mostly due to lengthy intervals between the development of goiter and presentation to the hospital. The three most common etiologies were toxic multinodular goiter (TMNG) (55.45%) toxic nodular goiter (TNG) (37.44%) and Graves’ hyperthyroidism (6.16%) (Fig. 1).

**Table 1:** Socio-demographic and clinical characteristics among hyperthyroid patients attending UoGCSH (*N* = 211)

Characteristics	
Mean age, years (±SD)	47.25 (±14.26)
Smoking, N (%)	
Yes	142 (67.30%)
No	68 (32.23%)
Data missing	1 (0.47%)
Sex, N (%)	
Male	12 (5.69%)
Female	199 (94.31%)
WHO goiter size, N (%)	
0	3 (1.62%)
I	48 (25.95%)
II	104 (56.22%)
III	30 (16.22%)
Mean duration of symptoms before seeking treatment, months (±SD)	88.23 (±138.35)
Mean SBP at diagnosis (±SD)	122.34(±20.0.6)
Mean DBP at diagnosis (±SD)	75.48 (±12.54)
Mean PR at diagnosis (±SD)	102.81 (±19.80)
Mean RR at diagnosis (±SD)	24.43 (±7.02)
Mean Body temperature at diagnosis, centigrade (±SD)	36.62 (±1.41)
FT4 values in pmol/l (*N* = 149) N (%**)**	
< 100	21 (14.09%)
100–200	49 (32.89%)
201–300	30 (20.13%)
≥ 300	49 (32.89%)
T3 values in nmol/l (*N* = 131) N (%)	
< 2.3	23 (17.56%)
2.3–5.0	48 (36.64%)
5.1–7.5	37 (28.24%)
7.6–10	17 (12.98%)
> 10	6 (4.58%)

*DBP* Diastolic Blood Pressure, *FT4* Free Thyroxine, *PR* Pulse Rate, *RR* Respiratory Rate, *SBP* Systolic Blood Pressure, *SD* Standard Deviation, *T3* Triiodo Thyronine, *WHO* World Health Organization

### Signs and symptoms and comorbidities

Majority of the patients experienced palpitation (83.41%), heat intolerance (70.14%) and increase sweating (55.6%) (Table 2). Hypertension was the most common comorbid condition which occurred in 3.76% (*N* = 8) of patients.

**Table 2:** Signs and symptoms thyrotoxicosis among hyperthyroid patients attending UoGCSH (*N* = 211)

Sign/symptoms (*N* = 211)	Number of patients (%)
Palpitation	176 (83.41%)
Heat intolerance	148 (70.14%)
Increased sweating	111 (52.61%)
Tachycardia	84 (39.81%)
Fatigue	80 (37.91%)
Warm moist skin	79 (37.44%)
Weight loss	57 (27.01%)
Increased appetite	31 (14.69%)
Irritability	29 (13.74%)
Dyspnea	22 (10.43%)
Hyperactivity	17 (8.06%)
Tremor	14 (6.64%)
Menstrual irregularities (*N* = 199-females only)	7 (3.52%)
Exophthalmos	4 (1.90%)
Nervousness	2 (0.95%)
Diarrhea	2 (0.95%)
Polyuria	1 (0.47%)
Staring appearance	1 (0.47%)

### Hospitalization for thyrotoxicosis and its complications

CHF and atrial fibrillation (AF) were the two most common complications of thyrotoxicosis which required patient hospitalization. Three patients also experienced thyroid storm (Table 3).

**Table 3:** Hospitalization for thyrotoxicosis and its complications among hyperthyroid patients attending UoGCSH (*N* = 211)

Reason for hospitalization	Frequency (%)
AF	2 (0.96%)
CHF	11 (5.24%)
CHF + AF	16 (7.62%)
Thyroid storm	3 (1.43%)
Total	32 (15.17%)

*AF* Atrial Fibrillation, *CHF* Congestive Heart Failure

### Current medication history

Because methimazole or carbimazole are not easily available locally, all of the 211 patients included in this study were taking PTU. The initial dosage of PTU in majority of the patients (*N* = 193) was 300 mg/d (*N* = 180) and followed by 200 mg/d (*N* = 13). The remaining 18 patients received PTU ranging from 50 mg/d (*N* = 1) to 800 mg/d (*N* = 3). The maintenance dosages of PTU were not any different from the initial dosages as 175 and 18 patients took 300 mg/d and 200 mg/d of PTU, respectively. The remaining patients took PTU at 50 (*N* = 1) to 600 mg/d (*N* = 2). Among beta-blockers, atenolol (*N* = 131) was the most common prescribed medication along with the PTU, whereas metoprolol (*N* = 3) was the least prescribed one. Among the medications used for the management of comorbidities, furosemide (*N* = 39) and spironolactone (*N* = 18) were the most common prescribed ones (Table 4).

**Table 4:** Current medications among hyperthyroid patients attending UoGCSH

Treatment modality	Frequency (%)
PTU	211 (100%)
Propranolol	94 (44.55%)
Metoprolol	3 (1.42%)
Atenolol	131 (62.09%)
Amlodipine	8 (11.76%)
Furosemide	39 (53.33%)
Spironolactone	18 (26.46%)
Captopril	1 (1.47%)
Aspirin	5 (7.35%)
Enalapril	8 (11.76%)
Digoxin	13 (19.11%)
Warfarin	8 (11.76%)
Salbutamol	3 (4.41%)
Antiretroviral therapy	2 (2.94%)
Verapamil	1 (1.47%)
Hydrochlorothiazide	9 (13.23%)

*PTU* Propylthiouracil

### Treatment outcomes

Majority of the patients (*N* = 188) had their symptoms resolved. The mean time to symptom resolution was 5.37 (±6.59) months. Sixty-two (29.81%) and 122 (58.65%) patients achieved normalization of TSH and FT4, respectively. The mean time to normalization of TSH and FT4 was 13 (±13.28) and 11.53 (±13.39) months, respectively. On the other hand, T3 and all three TFTs were normalized only among 79 (38.16%) and 55 (26.32%) patients, respectively. Only one (0.47%) patient experienced arthralgia as an adverse effect due to PTU, and no other adverse effect was recorded.

Univariate analysis identified that patients with TMNG had less chance of achieving normalization of TSH by 65.7% [Crude Hazard Ratio (CHR) (95% CI): 0.343 (0.141–0.836), *p* = 0.019] compared to patients with GD. However, this effect was not maintained up on multivariate analysis. On the other hand, older age and higher baseline FT4 levels were shown to modestly reduce the chances of achieving normalization of TSH (Table 5). Older age [CHR (95% CI): 0.983 (0.970–0.996), *p* = 0.009] and duration of symptoms before seeking treatment [CHR (95% CI): 0.998 (0.997–0.999), *p* = 0.047] also showed a statistically significant but modest effect on FT4 normalization. However, they failed to maintain this effect up on multivariate analysis (Table 6).

**Table 5:** Predictors of TSH normalization among hyperthyroid patients attending UoGCSH

Variables		Univariate analysis		Multivariate analysis	
		CHR (95% CI)	*p*-value	AHR (95% CI)	*p*-value
Age (years)		0.987 [0.970–1.005]	0.151	0.967 [0.937–0.998]	0.036
Gender	Female	0.992 [0.359–2.379]	0.988	–	–
Etiology	GD	1		1	
	TMNG	0.343 [0.141–0.836]	0.019	0.580 [0.147–2.287]	0.436
	Toxic nodular goiter	0.415 [0.166–1.038]	0.060	0.872 [0.218–3.499]	0.847
Duration of symptoms before treatment (months)		0.998 [0.996–1.000]	0.141	1.000 [0.997–1.002]	0.708
WHO goiter size	0	1		1	
	I	0.248 [0.030–2.024]	0.193	–	–
	II	0.356 [0.047–2.676]	0.315	–	–
	III	0.336 [0.042–2.692]	0.304	–	–
Baseline TSH		1.293 [0.891–1.874]	0.175	1.214 [0.647–2.279]	0.545
Baseline FT4		0.993 [0.990–0.996]	0.000	0.992 [0.988–0.997]	0.000
Baseline T3		0.907 [0.797–1.033]	0.142	0.966 [0.887–1.051]	0.418
PTU dose: initial		1.001 [0.999–1.004]	0.246	1.001 [0.999–1.004]	0.289
PTU dose: Maintenance		0.999 [0.995–1.004]	0.832	0.999 [0.994–1.004]	0.662

*AHR* Adjusted Hazard Ratio, *CHR* Crude Hazard Ratio, *CI* Confidence Interval, *GD* Grave’s Disease, *FT4* Free Thyroxine, *PTU* Propylthiouracil, *T3* Triiodo thyronine, *TMNG* Toxic Multinodular Goiter, *TFT* Thyroid Function Tests, *TSH* Thyroid Stimulating Hormone, *WHO* World Health Organization

**Table 6:** Predictors of FT4 normalization among hyperthyroid patients attending UoGCSH

	FT4 normalization		FT4 normalization	
	CHR (95% CI)	p-value	AHR (95% CI)	p-value
Age (years)	0.983 (0.970–0.996)	0.009	0.999 (0.974–1.004)	0.160
Duration of symptoms before treatment (months)	0.998 (0.997–0.999)	0.047	0.999 (0.997–1.000)	0.140
PTU dose: initial	1.002 (1.000–1.003)	0.108	1.001 (0.999–1.003)	0.447

*AHR* Adjusted Hazard Ratio, *CHR* Crude Hazard Ratio, *CI* Confidence Interval, *FT4* Free Thyroxine, *PTU* Propylthiouracil

## Discussion

Evaluation of management of thyrotoxicosis requires the periodic determination of the serum level of thyroid hormones. Despite the efforts made to measure these hormones in routine clinical practice, little is known about outcome of thyrotoxicosis management in Ethiopia. The primary outcomes of this research were to determine normalization of TFTs and time needed for normalization of these tests after initiation of ATDs. TSH measurement is very important for the determination of treatment outcome in hyperthyroidism because it is more specific and sensitive indicator than other TFT values [9]. Compared to all three TFTs most patients had normalization of TSH. But TSH normalization took longer period of time as compared to T3 and FT4. This is contrary to what other studies report. Previous studies revealed that thyroid hormone levels begin to drop in 2 to 3 weeks of starting ATDs. After 6 weeks of starting ATDs, 90% of patients with GD will be euthyroid but more than 24 months ATD treatment is usually necessary to normalize TFTs in patients with TMNG [10]. The high proportion of patients with TMNG in the present study may be the main reason for low TSH normalization rates. A prospective study reported the cumulative recovery rates of serum TSH among GD patients to be: 36.7% (33/90) at 3 months, 72.2% (65/90) at 6 months, 86.7% (78/90) at 9 months, 95.6% (86/90) at 12 months [11].

In the present study, age slightly increased rate of TSH normalization. Mohlin et al. found that age and gender had no significant relation for prognosis of the disease [12]. Martin et al. study also reported that GD patients, family and personal history of thyroid and autoimmune disorders, age at diagnosis, gender, goiter size, and ophthalmopathy presence did not influence the time to TSH recovery [11]. On the other hand higher baseline FT4 levels were shown to reduce rate of TSH normalization by 0.8%. Other than these two, none of the other factors showed a statistically significant effect on the TSH values. Upon univariate analysis older age (CHR = 0.983, *p* = 0.009) and duration of symptoms before seeking treatment (CHR = 0.998, *p* = 0.047) showed a statistically significant but modest effect on FT4 normalization. The mean duration of symptoms before seeking treatment was 88 months which is a very long time. It may indicate the knowledge and attitude of the society towards hyperthyroidism and may require awareness creation with regard to early medical seeking behavior. It showed that as patients’ age gets older, the rate of FT4 normalization decreased by 1.7%. Likewise, as duration of symptom before seeking treatment increased the rate of FT4 normalization was decreased by 0.2%. But, they failed to maintain this effect up on multivariate analysis. Mohlin et al. reported that palpable goiter, or according to WHO goiter size classification stage II goiter size, was associated with worse prognosis compared with no goiter (51.2 vs. 68.9% in remission after 5 years; *P* = 0.014 [12]. However, the present study did not identify WHO goiter size as independent predicator of FT4 normalization. Mohlin et al. study also found that previous smokers had a higher remission rate than either current smokers or non-smokers (85.7 vs. 55.8 vs. 50.5%) respectively, in remission after 5 years; *P* = 0.003) but there was no difference in prognosis between smokers and non-smokers [12]. On the other hand, the present study was not able to made relation with smokers and nonsmokers.

Though most patient had symptom resolution, it took longer time than what was reported by other studies. A retrospective multicenter study by Tagami et al. reported that the signs and symptoms of patients with hyperthyroidism showed a significant improvement after just 4 weeks of taking beta-adrenergic blocking agents [13].

Because of less frequency of administration and lower risk of adverse effects, MMI is recommended as preferred ATD over PTU and should be used among all patients except during the first trimester of pregnancy, in the treatment of thyroid storm or in patients with minor adverse reactions to MMI [1, 14–16]. However, all of the patients in the present study used only PTU. MMI is yet to be included in the national medicines formulary [17] and standard treatment guideline [18] of Ethiopia. As a result, availability of MMI remains a challenge rendering clinicians to rely mainly on the routinely available PTU.

If hyperthyroidism is left untreated, it can lead to cardiovascular complications. According to our study 11 (5.245%), 2 (0.96%) and 16 (7.62%) patients developed CHF, AF and CHF + AF, respectively. Another 3 (1.43%) patients also developed thyroid storm. A recent review article stated that the prevalence of AF among hyperthyroidism was 16–60% [19], which is more than what was found in the present study. On the other hand, Akamizu et al. found that the incidence of thyroid storm in Japan was estimated to be 0.20 persons per 100,000 population per year, accounting for 0.22% of all thyrotoxic patients and 5.4% of hospitalized thyrotoxic patients [20].

There are several adverse effects that may occur during ATD treatment. One meta-analysis found that minor complications such as a rash, gastric intolerance and arthralgia were observed in up to 19% of patients taking ATDs, while major complications occur rarely [21]. Minor allergic side effects, such as a limited, minor rash, may occur in up to 5% of patients taking either MMI or PTU [9]. In the present study, only one patient experienced arthralgia side effect (0.47%). However, as this was a retrospective study, it was difficult to objectively assess adverse effects.

### Strengths and limitations

The present study has several strengths. The major strength of this study was that it was the first study to evaluate treatment out come in hyperthyroid patients who is taking ATDs in Ethiopia. This makes it to be a corner stone for the upcoming researches. The study has tried to include almost all eligible subjects. However, the study is not without limitations. The major limitation of this study was that it was a retrospective study and relied on the data from the medical records. Large numbers of patient medical records were also missing which made to depend on smaller sample size. The current study did not assess remission and relapses. In addition, due to its retrospective nature of the study, we were unable to determine the level of adherence to treatment. Therefore, interpretation of the findings of this study should take this limitation in to consideration.

## Conclusions

Despite international guidelines’ recommendation of MMI, PTU was the only agent used for the management of hyperthyroidism. TSH and all TFT were normalized in only less than one-third of the participants. Though most patients achieved resolution of symptoms, it took longer period of time than the expected. The study showed that most of the patients were not able to achieve the state of euthyroidism within the recommended period of time. In order to identify the reasons for poor treatment outcomes, a large-scale, multicenter prospective study needs to be conducted giving emphasis mainly on medication adherence and adverse effects.

## Abbreviations

AF: Atrial fibrillation
ATDs: Anti-thyroid drug
BBs: Beta adrenergic receptor blockers
CHF: Chronic heart failure
FT4: Free thyroxin
GD: Graves’ disease
MMI: Methimazole
PTU: Propylthiouracil
T3: Triiodothyronine
TFT: Thyroid function test
TMNG: Toxic multinodular goiter
TSH: Thyroid stimulating hormone
UoGCSH: University of Gondar Comprehensive Specialized Hospital
WHO: World Health Organization
